# Ensemble Deep Learning Models for Brain Connectivity in Alzheimer's Disease Diagnosis via Different Perspectives

**DOI:** 10.1002/alz70856_097585

**Published:** 2025-12-24

**Authors:** Ke Chen, Ying Weng, Tom Dening, Akram A. Hosseini, Guokun Zuo

**Affiliations:** ^1^ University of Nottingham Ningbo China, Ningbo, Zhejiang, China; ^2^ University of Nottingham, Nottingham, United Kingdom; ^3^ Nottingham University Hospitals NHS Trust, Queens Medical Center, Nottingham, United Kingdom; ^4^ The Ningbo Institute of Industrial Technology (CNITECH) of the Chinese Academy of Sciences (CAS), Ningbo, China

## Abstract

**Background:**

Accurate diagnosis of Alzheimer's disease (AD) remains a significant challenge in neuroimaging research. Combining anatomical and structural connectivity features offers a promising avenue for improving diagnostic performance. This study introduces two advanced ensemble methods, Brain Ensemble Network (BrainEnsNet) and Population Ensemble Network (PopEnsNet), designed to leverage multimodal brain connectivity for AD diagnosis.

**Method:**

BrainEnsNet integrates anatomical features with brain connectivity graphs, optimizing individual sub‐model performance. PopEnsNet enhances this framework by constructing population‐level relationships and employing node correlation‐guided aggregation to achieve superior node classification. Performance was evaluated across multiple scales using metrics such as accuracy, precision, recall, and F1‐score.

**Result:**

With features derived from two scales of Lausanne2018 atlas, BrainEnsNet achieved accuracies of 80.82% (Scale 1) and 81.50% (Scale 2) for AD/Mild Cognitive Impairment (MCI)/Normal Cognitive (NC), consistently outperforming its sub‐models. PopEnsNet further improved diagnostic outcomes with accuracies of 81.40% (Scale 1) and 82.03% (Scale 2), demonstrating its effectiveness in incorporating population‐level relationships.

**Conclusion:**

The results highlight the potential of multimodal ensemble networks in neuroimaging‐based AD diagnosis. By integrating population‐level relationships and robust feature representations, BrainEnsNet and PopEnsNet set a foundation for advancing diagnostic accuracy. Future research could incorporate longitudinal data and additional imaging modalities to enhance clinical utility and generalizability.

